# The Value of Gude’s Pepto-Mangan in Anaemia

**Published:** 1903-07

**Authors:** Enrique Diago, Jose F. Benitez

**Affiliations:** Havana, Superintendent of Hospital No. 1, Havana, Cuba; Havana, Chief of the Laboratory, Hospital No. 1, Havana, Cuba


### The Value of Gude's Pepto-Mangan in Anaemia.

BY DR. ENRIQUE DIAGO, HAVANA,
Superintendent of Hospital No. 1, Havana, Cuba,

AND

DR. JOSE F. BENITEZ, HAVANA,
Chief of the Laboratory, Hospital No. 1, Havana, Cuba.

(Translated from the "Progreso Medico," Havana, April, 1902.)

Anæmia is a very common disease in this country (Cuba), and consequently one against which the physician is often obliged to contend in the practice of his art. While the use of the ordinary iron preparations often gives all the effects that could be desired, yet it usually producs a condition which may be regarded as a secondary disease—constipation. In looking about for a preparation which would not present this very serious disadvantage, which can not always be counteracted by the coincident administration of laxatives, we came across Gude's Pepto-Mangan, which, according to the published statements of many clinicians, seemed to us a remedy worth trial in a large series of cases. Accordingly, we obtained a sufficient supply of this preparation for our hospital, and began to treat all our cases of anæmia, in which iron was indicated, with Gude's Pepto-Mangan.

In presenting now the results of our observations with this pharmaceutical compound, we may say at once that our expectations were more than realized, when we noted its efficiency in combating the disease, and its perfect palatability and freedom from constipating after effects.

One of us, Dr. Benitez, chief of the laboratory of the hospital, undertook the task of keeping minute records of all the cases observed, including a record of the amount of hemoglobin and of the number of the red blood cells, both before and after the treatment. For the purpose of illustration, we relate briefly six cases, which show conclusively the effects of Gude's Pepto-Mangan on persons with anæmia, and prove without doubt that the administration of this remedy is connected with none of the disadvantages and discomforts attending the use of the ordinary preparations of iron.

Case 1.—N. G., aged 26 years, was admitted to the hospital, suffering from loss of nutrition, emaciation, pallor of the skin and mucous membranes, loss of memory, anorexia, mental depression,—a word, from all the typical symptoms of anæmia. This condition was traced in his case to a chronic malaria, from which the patient had been suffering for a long time. The patient weighed only 102 pounds at the time of admission.

Pepto-Mangan (Gude) was given in doses of two tablespoonfuls twice daily, at breakfast and at dinner, respectively, with some cinchona wine. The first blood examination showed 2,400,000 red blood corpuscles c. m., by the Thoma-Zeiss method. Ten days after the beginning of the treatment, this patient, who had been so extremely pale when he entered, began to improve as regards the color of his cheeks and general appearance. His general well-being was so marked that he spoke with pleasure of the marked improvement in his condition which had taken place since he had been taking the new remedy at our hospital. In these ten days he had gained five pounds in weight and was able to walk around the ward without the lassitude which he had felt when he was admitted. The blood was examined a second time, showing an increase of 300,000 red blood cells. The patient was discharged cured after fifty days' treatment, weighing 130 pounds and with a blood-count indicating 2,800,000 red blood cells c. m.

Case 2.—Mrs. C. D., aged 34 years, who gave a history of miscarriage, was admitted with the symptoms of anæmia, secondary to the loss of blood occasioned by the accident mentioned. The chief symptoms were emaciation, loss of strength, and gastro-intestinal disturbances. She weighed only 90 pounds when she entered the hospital, and her blood showed a marked diminution in the amount of hemoglobin, and only 2,300,000 red blood cells to the cubic millimetre.

Gude's Pepto-Mangan was prescribed in the same doses as in the preceding case, and all went well until the tenth day, when the patient of her own accord, in order to facilitate the cure, and to accelerate the recovery, took five tablespoonfuls of the preparation during the day, causing a slight disorder of the stomach. The administration of Pepto-Mangan was thereupon discontinued, and tablets of bismuth and salol, together with a purgative, were given. Five days later, the Pepto-Mangan was resumed, at first in doses of two teaspoonfuls, and two days later in doses of two tablespoonfuls. The further course of the treatment went on without any mishap, and the patient recovered completely. On leaving the hospital the hemoglobin was found normal, and the number of red blood cells was found to have increased to 3,500,000 c. m., while the patient's weight had increased twenty-one pounds within fifty days.

Case 3.—Mr. M. D., aged 26 years, who had suffered during the preceding month from an attack of acute articular rheumatism involving a number of joints, entered the hospital complaining of the symptoms of anæmia. He had the appearance of a convalescent, with pale skin and mucous membranes, fatigue in walking, emaciation, etc. There was edema about the ankles, but no valvular lesion in the heart, and there were in addition, absence of appetite, insomnia, functional depression of the genital apparatus, and dyspepsia. The patient weighed only 92 pounds, and his blood when examined showed a decrease in the amount of hemaglobin and only 2,500,000 red blood cells c. m. At the end of fifteen days' treatment, which consisted of the administration of two tablespoonfuls of Pepto-Mangan (Gude) at breakfast, of the same amount at dinner and of an additional tablespoonful at noon, the patient had gained a great deal of strength, his pallor had almost disappeared, the hemoglobin had increased and reached its normal quantity, and the red blood cells had increased to 3,200,000 c. m. The patient was therefore discharged completely cured at the end of forty days after admission.

CASE 4.—Mr. R. G., aged 42 years, who did not show any signs of organic disease, and who presented no characteristics of a gouty or lithemic diathesis, was admitted to the hospital in a greatly disturbed state of mind on account of attacks of vertigo, palpitation of the heart, extreme weakness, and various erratic pains in the muscles. He gave a history of a recent attack of influenza, during which his nervous symptoms had become intensified. He had not had a very marked rise of temperature, and the respiratory passages were scarcely affected during this attack, but there were severe pains in the back and joints, and an intense headache. The examination of the blood showed the presence of 3,000,000 red blood cells c. m., and the patient was found to weigh only 110 pounds.

He was placed exclusively on Pepto-Mangan (Gude) treatment. Twenty days later, the pains had ceased; he ate well; his weight had increased to the extent of four pounds, and the red blood corpuscles had increased in number by 200,000. Thirty days after admission he was discharged cured.

CASE 5.—Miss C. P., aged 16 years, was admitted to the hospital with a very pale skin and a deficient muscular and adipose development. Her menstruation had become irregular, and she had suffered from various nervous disturbances. Her growth had not kept in harmony with her nutrition, and she presented the characteristics of chloro-anæmia, as frequently seen in Cuban girls, namely, accompanied by a series of neurasthenic symptoms. She weighed only 87 pounds, and the blood count showed only 1,800,000 red blood corpuscles c. m. After ten days' treatment, the number of red blood corpuscles increased by 200,000, and the weight of the patient by three pounds. Twenty-six days after admission, she was removed from the hospital by her relatives, and on discharge her weight was 94 pounds.

CASE 6.—Mr. G. F., aged 38 years, whose previous history was negative, and who had not suffered from any severe illness shortly before admission, entered complaining of loss of flesh and strength, decrease of normal weight and extraordinary fatigue after his usual work. He attributed these symptoms to transgressions of hygienic rules. The first blood examination showed 2,600,000 red blood cells c. m. The patient weighed 106 pounds on admission. Thirty-six days later, after having been under treatment with Pepto-Mangan (Gude) during the entire period, he was discharged at his own request. He had increased eleven pounds in weight and his red corpuscles numbered 2,850,000 c. m. (an increase of 250,000). He went back to his usual work without experiencing any unusual fatigue.

To sum up the results obtained with the employment of Pepto-Mangan (Gude) in the treatment of anæmias, we may say, conscientiously, that it is the best remedy we know of for this purpose, and that we do not hesitate to commend it to the medical profession at large, and especially to our confreres in Cuba, as an iron preparation that possesses all the advantages that can be demanded of such a remedy and none of the disadvantages that are characteristic of other iron preparations. We would especially emphasize also that Pepto-Mangan (Gude) is very pleasant to the taste, and is most easily taken by patients of all ages and with the most delicate digestions.

Havana, March, 1902.

